# Social Network Factors Associated with the Intention to Use Digital Mental Health Interventions Among AANHPI Emerging Adults: Application and Integration of the Network Episode Model

**DOI:** 10.21203/rs.3.rs-9832200/v1

**Published:** 2026-06-30

**Authors:** John Bunyi, Shinyi Wu, Angel Hsing-Chi Hwang, Hans Oh

**Affiliations:** University of Southern California; University of Southern California; University of Southern California; University of Southern California

**Keywords:** digital mental health interventions, AANHPI, help-seeking, Network Episode Model, social networks, stigma, emerging adults, logistic regression

## Abstract

**Background:**

Asian American, Native Hawaiian, and Pacific Islander (AANHPI) emerging adults face significant mental health disparities yet consistently underutilize formal mental health services. Digital mental health interventions (DMHI) have been proposed as a solution; however, the social network factors shaping intention to use DMHIs in this population remain poorly understood among this highly collectivist population. Prior work has established that individual-level factors derived from the Seeking Mental Health Care model (i.e., perceived need, mental health literacy, and stigma) are associated with intention to use DMHI among AANHPI emerging adults. The present study integrates the Network Episode Model to examine whether social network characteristics are associated with DMHI intention and whether they moderate established associations with individual-level factors.

**Methods:**

Cross-sectional survey data were drawn from a Qualtrics panel study of AANHPI emerging adults ages 18–29 (N = 1,577). Nested multivariable logistic regression models were estimated: Model 1 replicated the individual-level specification; Model 2 added social network variables; and Models 3a–3c each tested a theoretically motivated interaction term. A Bonferroni-corrected threshold of α = .017 was applied across the three interaction tests.

**Results:**

Adding social network variables significantly improved model fit. Awareness that friends or family use DMHIs and current therapy engagement were strongly associated with DMHI intention. Structural network size and social support quality did not reach significance. All individual-level variables remained significant after social-network variable inclusion. Symptom severity was non-significant across all models. Only the friends and family DMHI use × public stigma interaction was significant: among participants uncertain whether their network used DMHIs, higher public stigma was associated with substantially greater DMHI intention. Significant ethnic heterogeneity persisted across all models.

**Conclusions:**

Knowledge of peer DMHI use and formal treatment engagement, contribute to DMHI intention beyond individual-level factors, supporting an integrated individual and social network framework. The significant stigma × peer DMHI use interaction suggests stigma may suppress network conversations about digital help-seeking, pointing to distinct intervention approaches for this subgroup. Findings underscore the importance of network-level strategies for increasing DMHI engagement in AANHPI communities.

## INTRODUCTION

### AANHPI emerging adults, Mental illness, and help-seeking

Asian American and Native Hawaiian/Pacific Islander (AANHPI) emerging adults (ages 18–29) face rates of mental illness that are comparable to or worse than the general population in the United States (1, 2, 3, 4), with over 30% of AANHPI individuals ages 18–25 experiencing some form of mental illness in 2024 (4) and with rates of serious mental illness and suicide on the rise (5, 6). Yet, AANHPI individuals consistently underutilize formal mental health care compared to the general population (8.6% of Asian Americans vs. 17.9% of the general population) (7). These patterns of limited help-seeking could be due to numerous factors, such as stigma around mental illness, the “model minority” stereotype, limited mental health literacy, a lack of culturally responsive providers, a preference for “informal” supports such as family or other community members, and various norms rooted in AANHPI culture. Thus, mental health researchers have sought new ways to reach AANHPI emerging adults, leveraging developing technologies in a generation that has been highly connected to digital devices and the Internet.

### Digital Mental Health and AANHPI

Digital mental health (DMHIs) has been touted as a potential solution to address a number of barriers to help-seeking and access to mental health care (8, 9, 10, 11). For AANHPI emerging adults, who are cited as a “digitally native” group more likely to be comfortable using digital technology for health care (12), DMHIs present a particularly interesting solution given the alignment between the needs that DMHIs aim to address, and the barriers faced by AANHPI (13, 14, 15). In fact, a number of studies have begun to explore culturally tailored digital interventions for AANHPI (16, 17). However, little is known about the drivers of the intention to use DMHIs or to seek help digitally among AANHPI, resulting in a limited understanding of the pathways of help-seeking among AANHPI.

### Leveraging existing help-seeking models for understanding “Digital Help-Seeking”

Existing research on DMHI and the development of such tools is largely atheoretical with even fewer being informed by health behavior models (18). Research that is driven by behavior theories often focus on individual-level factors such as technology acceptance, cues to action, or attitudes to a behavior. While such frameworks are helpful in understanding the decision to utilize DMHIs, they are limited in that they tend to view help-seeking as a discrete individual decision, abstracted from social environments in which distress and help-seeking occurs.

A recent study from this research team provides a relevant foundation that examined how individual-level factors are associated with intention to use DMHI among the same sample of AANHPI emerging adults (19). Using the Seeking Mental Health Care Model (SMHC) (20) as a framework, the study found that *perceived need for support*, *mental health literacy*, *public stigma* (i.e., the perception that the public views those with mental illness negatively), and *self-stigma* (i.e., internalized stigma), were each associated with the intention to use DMHIs. These findings confirm that individual-level and attitudinal factors are meaningfully associated with digital help-seeking and intention to use DMHIs.

Additionally, significant ethnic heterogeneity was also observed, with Filipino, Indian, Japanese, and Self-Described AANHPI subgroups showing higher odds of intention to use DMHIs relative to Chinese participants, drawing attention to the importance of recognizing differences between AANHPI ethnicities, and how they perceive digital tools for mental health. Moreso, these differences between ethnic groups likely reflect unique sociocultural experiences, norms (15), patterns of technology use (21), and levels of acculturation (22), which is reflected in differing tendencies of help-seeking for mental health (15). While the SMHC is useful in framing how individual-level factors affect help-seeking, it does not account for social-level factors such as networks of informal support, sources of instrumental support, formal treatment networks, and whether others in individuals’ networks have themselves used DMHIs. This gap is consequential, as mental health help-seeking among AANHPI populations is known to be deeply embedded in family and community dynamics that individual-level models alone cannot capture (15, 23).

### Social networks, social support, help-seeking, and digital mental health

Despite recognition that help-seeking for mental health is a socially embedded process, DMHI research has devoted comparatively little attention to the role of interpersonal networks and social support in shaping the decision to seek digital care. This is despite the extensive evidence from broader help-seeking literature that social networks and social support influence mental health care utilization in multiple ways (24, 25, 26).

*Social support* is consistently associated with help-seeking for mental health, albeit in complex and sometimes contradictory ways. Supportive relationships can sometimes proxy for formal treatment leading to a delay in formal treatment seeking (26). However, social support can also provide a push to enter treatment for those with more severe mental health disorders (25, 26).

*Social networks* often play an active role in prompting individuals to seek professional help through direct encouragement and information sharing (24, 25, 26). Relatedly, network actors may also play a role in modeling mental health help-seeking behaviors (24, 25) helping to normalize help-seeking.

These dynamics are amplified among AANHPI communities, where informal networks of support such as family, community, and religious leaders are key sources of mental health support (15). Such a reliance on informal networks of support reflects a cultural strength in that such networks can be a direct source of support for mental health or a source of information, but such networks also delay formal treatment utilization. Given the dearth of research in DMHIs and social networks, the role of such networks in influencing DMHI utilization remains largely unknown, and whether social networks support or hinder DMHI use and digital help-seeking becomes an empirical question with direct implications for intervention delivery.

Thus, the present study leverages the Network Episode Model (NEM) (24), a sociological framework that conceptualizes help-seeking not as a discrete individual decision but as an ongoing, socially negotiated process embedded within personal networks. The NEM posits that when individuals experience a mental health episode, their care pathway is substantially affected by what people in their network, known as network actors, believe about mental illness, who they talk to in their network, and whether network actors have previously engaged with formal or informal care (27). Unlike social support (i.e., emotional, informational, or instrumental resources), social networks refer more to the size, composition, and pattern of an individual’s social ties (28). Critically, the NEM argues that both dimensions matter for help-seeking, with network structure shaping who an individual can reach and how information about care travels through a social environment, while support quality shapes whether those ties are experienced as resources that enable or discourage action (24, 27). The NEM positions social ties as active mechanisms through which illness is defined, normalized, or minimized, and through which help-seeking behavior is channeled or discouraged. This framing is particularly relevant for AANHPI emerging adults for whom family systems often serve as the primary context for understanding and responding to psychological distress (29). Within many AANHPI cultural frameworks, mental health concerns are not experienced or disclosed as individual problems but are filtered through relational obligations, family reputation, and collective well-being. By combining the SMHC and NEM frameworks, we can more deeply understand the pathway through which DMHI intention forms.

### An integrated model of digital help-seeking

While the SMHC and NEM frameworks have each informed mental health help-seeking research independently, few studies have examined how individual-level attitudinal factors and social network characteristics operate *jointly* to shape help-seeking intention, and none have done so in the context of DMHI among AANHPI emerging adults. This study seeks to explore how these two frameworks can be complimentary, and perhaps shape a more comprehensive understanding of how people seek mental health support digitally. One possibility, as suggested by Green and Pescosolido (30), is that larger and more robust social networks may compensate for lower individual-level perceived need, such that larger networks may offer a richer set of help-seeking options, and network actors may prompt individuals to seek support or even provide support themselves. Another possibility is that network actors could “model” help-seeking, as part of a phenomenon referred to as “experiential homophily” in which individuals are more likely to interact with and seek advice from others who have gone through similar health experiences (27). Furthermore, recognizing mental health problems and knowing where to seek support (i.e., mental health literacy) remains a daunting task (30). Because of this, the NEM suggests that individuals are likely to rely on and be exposed to the varying levels of knowledge and cultural beliefs of those in their social network to help define and respond to symptoms.

Like the SMHC, the NEM also recognizes the important and complex role that stigma plays in help-seeking (24, 27). As established in our prior work, public stigma demonstrated a positive association with DMHI intention, in which those with higher perceived public stigma were more likely to intend to use DMHIs. The NEM suggests that whether and how stigma shapes help-seeking is itself conditioned by the social environment and what an individual perceives others in their network to believe and do with respect to mental health care (27).

### Aims and Hypotheses

The present study has two primary aims. First, we examine whether social network characteristics, NEM variables (i.e., family and friend network size, perceived social support availability, current therapy use, and awareness of DMHI use among friends and family), are associated with intention to use DMHI among AANHPI emerging adults after accounting for established individual-level variables of help-seeking. Second, we seek to test a series of theoretically motivated interaction terms to explore whether social network factors moderate the associations between SMHC factors and DMHI intention. With respect to the first aim, we hypothesize that awareness of DMHI use among friends and family and current therapy use will be positively associated with DMHI intention (H1), consistent with the NEM’s emphasis on network-level drivers of help-seeking behavior. We further hypothesize that the size of friend and family networks will be more positively associated with DMHI intention (H2), reflecting a greater likelihood of finding network actors to receive advice from for help-seeking. Furthermore, we hypothesize that social support availability will be associated with the intention to use DMHIs (H3). Given the exploratory nature of the interaction tests, interaction models are treated as hypothesis-generating rather than confirmatory, and findings are interpreted accordingly. Across all models, we expect that the individual-level SMHC variables will remain robust to the inclusion of social network variables, reflecting the complementary rather than redundant contributions of the two frameworks.

## METHODS

### Study Design and Participants

Sampling and recruitment. This study utilized data from the Personalized Normative Feedback (PNF) Project (PI: Dr. Hans Oh; University of Southern California IRB: APP-24–05114), a cross-sectional online survey of Black and AANHPI emerging adults across the United States. A full description of the study design, sampling procedures, and recruitment methodology is provided in Bunyi et al. (19). A summary is provided here for context.

Data were collected via Qualtrics survey panels from July 2024 to September 2024. Qualtrics survey panels consist of pre-enrolled participants who consent to be contacted for research studies (Qualtrics, Provo, UT). To ensure sample quality and mitigate risks associated with online panel recruitment, multiple verification procedures were implemented prior to and during data collection, including CAPTCHA screening, vendor-applied identity confirmation protocols (TrueSample, Verity, SmartSample, USPS address verification, and digital fingerprinting), and cross-verification of respondent demographic information, residential addresses, and email accounts (31). Data were collected across three vendor sources, which are controlled for in all analyses. Participants provided informed consent electronically prior to survey initiation and were compensated with rewards appropriate to their recruitment pathway. We counted only participants who completed the full survey toward recruitment quotas.

Participants. The present study focuses on the AANHPI subsample of the PNF Project. To ensure adequate representation across the largest AANHPI ethnic subgroups, recruitment quotas were set with Qualtrics to obtain approximately 200 participants each from seven ethnic groups: Chinese, Filipino, Indian, Japanese, Korean, Native Hawaiian and Pacific Islander (NHPI), and Vietnamese, with an eighth open-ended “Other AANHPI” category, henceforth referred as “Self-Described AANHPI”. Eligibility criteria included self-identification as AANHPI, age between 18 and 29 years, and current residence in the United States. Following data collection, participants who identified with multiple AANHPI ethnic groups were recategorized as “Multi-Ethnic,” and those who identified as both AANHPI and another racial group were recategorized as “Multi-Racial AANHPI,” to better account for the distinct mental health experiences of individuals with multiple ethnic and racial backgrounds (14). After cleaning and recategorization, the final analytic sample consisted of 1,577 self-identified AANHPI individuals.

### Measures

The present study largely retained the measures from Bunyi et al. (19) and extended the measurement model by adding social network constructs derived from the NEM. A full psychometric description of the SMHC measures is provided in that prior work while key details and changes are summarized below for completeness. All measures were administered as part of the same Qualtrics survey instrument. Table 1 presents descriptive statistics for all study variables.

#### Outcome Variable.

The primary outcome variable was intention to use DMHIs, measured using a single dichotomous self-report item (yes/no) indicating whether the respondent intended to use a digital mental health tool. DMHIs were defined broadly to encompass a range of digital modalities, including wellness apps or websites, apps or websites designed to treat depression, anxiety, or other mental health conditions, online or telephone-based therapy services, and online support groups. This operationalization is consistent with recent calls in the literature for broad, inclusive definitions of DMHIs that reflect the range of technologies available to real-world users (32, 33, 34).

#### SMHC Variables.

Four individual-level variables derived from the SMHC model (20) were retained from the companion analysis. *Perceived need* for support was assessed using a single binary self-report item drawn from the California Health Interview Survey (35), indicating whether the respondent had felt a need for emotional or mental health support in the past 12 months (yes/no). *Mental health literacy (MHL)* was measured using a reduced 7-item scale adapted from the 26-item Mental Health Literacy Measure (36), assessing knowledge-oriented and resource-oriented literacy. Items were dichotomized and summed into a composite score, with higher scores indicating greater mental health literacy (α = 0.76). *Perceived public stigma* and *perceived self-stigma* were each derived from the 24-item Perceived Devaluation and Discrimination Scale (PDDS) (37), treated as continuous mean scores. Items 1, 2, 3, 4, 8, 10 were reverse-coded, and all item scores were totaled then averaged such that higher values indicated lower perceived stigma (public stigma α = 0.74; self-stigma α = 0.83).

#### NEM Variables.

Four social network constructs derived from the NEM were added for the present analysis. *Friends and family use of DMHI* was operationalized as a three-category variable reflecting the respondent’s awareness of DMHI use among their friends or family members: “yes” (confirmed awareness), “not known” (uncertain), or “no” (confirmed absence; reference category). This three-category operationalization was retained rather than dichotomized to preserve the theoretically and empirically meaningful distinction between confirmed absence of peer DMHI use and uncertainty about peer DMHI use, as these conditions may carry distinct implications for experiential homophily and stigma-related help-seeking dynamics (27). *Social network size* was assessed using the Lubben Social Network Scale-6 (LSNS-6) (38), a validated 6-item measure of social engagement with family and friends. The LSNS-6 yields a 3-item family subscales a 3-item friend subscale which were scored and entered separately as continuous variables to allow for differential associations between family and friend network size and DMHI intention, consistent with theoretical distinctions between family-centered and peer-centered help-seeking norms in AANHPI populations (7). Higher scores on each subscale indicate larger and more active social networks. *Perceived social support availability* was measured using a 6-item Medical Outcomes Study Social Support Survey (MOS) (39), a validated measure of functional social support encompassing emotional, informational, tangible, and affectionate support. The MOS total score was used as a continuous variable, with higher scores indicating greater perceived support availability. *Current therapy utilization* was assessed using a dichotomous yes/no question reflecting involvement in formal mental health treatment and thus having ties to formal care systems.

#### Covariates.

Sociodemographic covariates retained from the companion analysis included *sex* (dichotomous; female vs. male), *age* (continuous, in years), *educational attainment* (dichotomous; college degree or higher vs. less than college degree), *employment status* (dichotomous; currently working vs. not working), *health insurance status* (dichotomous; insured vs. uninsured), and *acculturation*, operationalized as a dichotomous proxy based on language spoken at home during childhood (mostly or only English vs. equally or predominantly non-English), consistent with the approach used in the companion analysis. *Ethnicity* was included using dummy-coded indicator variables with the Chinese subgroup serving as the reference category, yielding nine contrast groups: Filipino, Indian, Japanese, Korean, NHPI, Vietnamese, Self-Described AANHPI, Multi-Ethnic, and Multi-Racial AANHPI. Symptom severity was assessed using the Generalized Anxiety Disorder 7-item scale (GAD-7) and the Patient Health Questionnaire 9-item scale (PHQ-9). Given the substantial collinearity observed between these two instruments (r = .85), they were combined into a single four-category symptom severity variable rather than entered as separate continuous covariates: 0 = no clinically significant symptoms on either scale, 1 = depression only (PHQ-9 ≥ 10, GAD-7 < 10), 2 = anxiety only (GAD-7 ≥ 10, PHQ-9 < 10), and 3 = comorbid depression and anxiety (both ≥ 10). Standard clinical cutoffs of ≥ 10 were applied for both instruments to indicate levels of depression and anxiety that were moderate-to-severe (40, 41). This variable was dummy-coded with the no clinical symptoms category as the reference. A dummy-coded variable for vendor source (three levels; Vendor 1 as reference) was included in all models to account for systematic differences in survey response patterns across recruitment panel sources, consistent with the companion analysis.

### Analysis

Multivariable logistic regression was used to estimate the associations between individual-level SMHC variables, NEM variables, and the intention to use DMHIs among AANHPI emerging adults. Analyses were conducted using Stata Statistical Software Release 19 (42). Analyses proceeded in four stages: descriptive statistics, nested model building, interaction model testing, and marginal effects estimation for interactions of interest.

#### Descriptive statistics.

Descriptive statistics and Pearson correlations were computed to summarize the data and assess preliminary relationships between independent variables and the outcome variable prior to hypothesis testing. There was no missingness in the data, consistent with data collection methods. See Table 1 for Descriptives.

Model building and nested logistic regressions. A series of nested logistic regression models were estimated to examine the independent and additive contributions of SMHC and NEM predictors to DMHI intention. Model 1 replicated most of the SMHC-only model from the companion analysis, including perceived need, mental health literacy, public stigma, self-stigma, alongside symptom severity (replaced with a four-category variable as described above), all sociodemographic covariates and vendor source indicators. Model 2 extended Model 1 by adding the full set of NEM predictors (i.e., friends and family DMHI use, LSNS-6 family subscale, LSNS-6 friend subscale, MOS total score, and current therapy engagement) alongside all SMHC predictors and covariates from Model 1. A separate NEM-only model was estimated to examine the independent contributions of social-network variables (see Appendix A for a table presenting the NEM-only analysis). Variance inflation factors (VIF) were examined in the fully adjusted Model 2 to assess multicollinearity among predictors. Results are reported in Appendix B.

#### Interaction model testing.

Three theoretically motivated interaction models were estimated, each building on the full Model 2 specification, to test if social network variables moderate associations between SMHC variables and DMHI intention. Consistent with established recommendations for interaction testing in logistic regression (43), a single interaction term was entered per model alongside all Model 2 main effects and covariates, rather than entering multiple interaction terms simultaneously, to preserve statistical power and avoid model overfitting. Model 3a tested an interaction between LSNS-6 family subscale score and perceived need. Model 3b tested an interaction between friends and family DMHI use and mental health literacy. Model 3c tested the interaction between friends and family DMHI use and public stigma.

Prior to interaction term testing, all continuous predictors involved in interaction terms were mean-centered by subtracting the sample mean from each observed value. This reduces structural multicollinearity between main effects and their constituent interaction terms and improves the interpretability of main effect coefficients, which in mean-centered models reflect the association between a predictor and the outcome at the mean of the moderating variable rather than at its zero point (44).

To account for the increased risk of Type I error arising from multiple simultaneous interaction tests, a Bonferroni-corrected significance threshold of α = .017 (α = .05 / 3 interaction tests) is reported alongside conventional unadjusted p-values for all interaction terms. Null or non-significant interaction tables are included in the appendices.

#### Marginal effects estimation.

For interaction terms that were statistically significant, adjusted predicted probabilities were estimated and marginal effects plots were generated to facilitate interpretation of the interaction pattern. Predicted probabilities were computed at, one standard deviation below the mean, at the mean, and one standard deviation above the mean for continuous moderators, holding all other covariates at their observed values. For categorical moderators such as friends and family DMHI use, predicted probabilities were computed separately for each category.

Changes in model fit between Models 1, 2, and 3 (a/b/c) were evaluated using likelihood ratio tests and comparing AIC/BIC metrics, to assess the value of adding NEM predictors beyond the individual-level SMHC framework. All models were estimated using the full analytic sample (N = 1,577).

## RESULTS

### Sample Characteristics and Bivariate Correlations

Of the 1,577 AANHPI emerging adults in the analytic sample, 908 (57.6%) endorsed an intention to use DMHIs. The sample had a mean age of 24.6 years (SD = 3.1) among those endorsing intention compared to 23.1 years (SD = 3.4) among those not endorsing intention (t = −9.04, p < .001). Participants who endorsed intention were more likely to hold a college degree or higher (70.7% vs. 48.6%, χ^2^(1) = 79.50, p < .001), be currently employed (65.1% vs. 50.1%, χ^2^(1) = 35.82, p < .001), be insured (95.2% vs. 88.2%, χ^2^(1) = 26.0, p < .001), and report high acculturation (68.3% vs. 47.1%, χ^2^(1) = 71.70, p < .001). Males comprised 46.6% of the DMHI intention group compared to 53.4% of the non-intention group (χ^2^(1) = 11.54, p = .001). Full descriptive statistics are presented in Table 1.

### Multivariable Logistic Regression

Model 1 replicated the SMHC-only analysis from the companion paper, with symptom severity operationalized as a four-category covariate in place of separate continuous GAD-7 and PHQ-9 scores to address the substantial collinearity observed between those instruments (r = .85; see Appendix B for VIF results). Perceived need for support (AOR = 1.94, 95% CI [1.49, 2.53], p < .001), mental health literacy (AOR = 1.14, 95% CI [1.07, 1.22], p < .001), self-stigma (AOR = 1.38, 95% CI [1.07,1.78], p = .014) and public stigma (AOR = 0.60, 95% CI [0.44, 0.81], p = .001) each remained significantly associated with DMHI intention, consistent with findings from the original SMHC analysis. Among ethnic subgroups, Filipino (AOR = 1.75, p = .016), Indian (AOR = 1.79, p = .008), Japanese (AOR = 3.41, p < .001), and Self-Described AANHPI (AOR = 1.77, p = .020) participants showed significantly higher odds of DMHI intention relative to Chinese participants. Notably, none of the symptom severity categories reached significance relative to the no-symptoms reference group (depression only: AOR = 1.01, p = .946; anxiety only: AOR = 0.68, p = .194; comorbid: AOR = 1.12, p = .431), indicating that symptom profile did not independently predict DMHI intention once other factors were controlled. Full results are presented in Table 2.

Model 2: SMHC + NEM Main Effects Model. Adding the NEM predictors in Model 2 significantly improved model fit over Model 1 (LR χ^2^(5) = 39.96, p < .001), an incremental improvement consistent with the NEM predictors contributing meaningful explanatory value beyond individual-level SMHC factors. The full model was statistically significant (LR χ^2^(30) = 506.31, p < .001).

Among the NEM predictors, knowing friends or family who use DMHIs was strongly and positively associated with DMHI intention (AOR = 1.79, 95% CI [1.35, 2.39], p < .001). Uncertainty of network use of DMHIs, or the “not known” category was non-significant (AOR = 0.75, 95% CI [0.55, 1.03], p = .074). The LSNS-6 family subscale score did not reach significance (AOR = 1.03, 95% CI [0.99, 1.08], p = .126), and neither did the LSNS-6 friend subscale score (AOR = 0.99, p = .637). The MOS total score (AOR = 1.02, p = .153) was also non-significant. Current therapy engagement remained strongly associated with DMHI intention (AOR = 1.84, 95% CI [1.33, 2.55], p < .001).

All four SMHC predictors remained in their expected directions after the addition of NEM variables, with perceived need (AOR = 1.91, p < .001), mental health literacy (AOR = 1.11, p = .003), self-stigma (AOR = 1.41, 95% CI [1.09, 1.84], p = .010), and public stigma (AOR = 0.63, p = .002) retaining significance in Model 2. AIC improved from 1,735.47 (Model 1) to 1,705.52 (Model 2) and BIC decreased from 1,874.92 to 1,871.78, further confirming the improved fit of the expanded model. Full results are presented in Table 3.

Interaction Models (Models 3a–3c). Three theoretically motivated interaction models were estimated, each adding a single interaction term to the full Model 2 specification. Given the simultaneous testing of three interactions, a Bonferroni-corrected significance threshold of α = .017 is reported for the three interactions, alongside conventional unadjusted p-values. Non-significant interactions are presented in Appendices C and D.

Model 3a: Perceived Need × LSNS-6 Family. The interaction between perceived need and mean-centered LSNS-6 family subscale score did not reach statistical significance under either the Bonferroni-corrected or conventional threshold (AOR = 1.06, 95% CI [0.99, 1.13], p = .114; LR χ^2^(1) = 2.51, p = .113). All main effects from Model 2 were preserved in direction and approximate magnitude.

Model 3b: FF DMHI Use × Mental Health Literacy. The interaction between friends/family DMHI use and mean-centered mental health literacy was not statistically significant for either the “yes” category (AOR = 1.11, 95% CI [0.96, 1.29], p = .156) or the “not known” category (AOR = 1.14, 95% CI [0.97, 1.35], p = .112). Notably, the main effect of mental health literacy was no longer significant in this model (AOR = 1.04, p = .380) compared to Model 2.

**Figure 1:**
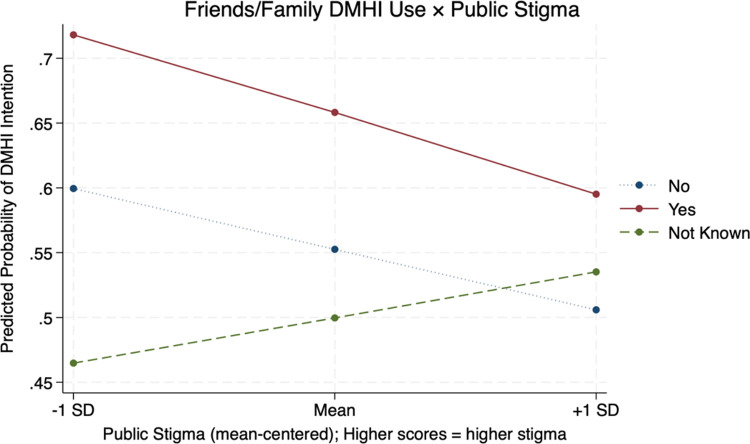
Adjusted predicted probabilities of DMHI intention by friends/family DMHI use status across levels of perceived public stigma (Model 3c).

Model 3c: FF DMHI Use × Public Stigma. The interaction between friends/family DMHI use and mean-centered public stigma was statistically significant for the “not known” category (AOR = 3.12, 95% CI [1.46, 6.66], p = .003), and remained significant after Bonferroni correction (α = .017). The “yes” category was not statistically significant (AOR = 0.79, 95% CI [0.41, 1.53], p = .486). The model fit improved meaningfully relative to Model 2 (LR χ^2^(2) = 12.65, p = .002; pseudo-R^2^ = 0.241 vs. 0.236; ΔAIC = −8.65; ΔBIC = +2.08), with the AIC favoring Model 3c over the main effects model. The main effect of public stigma strengthened in this model (AOR = 0.52, 95% CI [0.34, 0.82], p = .004) compared to Model 2. Adjusted predicted probabilities are plotted in Figure 1, which illustrates the diverging trajectories of DMHI intention across FF DMHI use categories as public stigma decreases. All primary SMHC predictors remained significant in Model 3c. Full results for Model 3c is presented in Table 4.

A summary of model fit statistics across all five models is presented in Table 5.

## DISCUSSION

This study examined individual-level and social network predictors of intention to use DMHIs among a large, ethnically diverse sample of AANHPI emerging adults, integrating the SMHC model with the NEM. Several key findings emerged across the nested model sequence and warrant discussion.

### The Role of Network DMHI use: NEM and Social Modeling

Across all models, there was a strong positive association between *knowing* friends and family who use DMHI and the intention to use DMHI, as was posited in hypothesis 1. This effect persisted at meaningful magnitude across models, remaining significant even after full adjustment for individual-level SMHC predictors, structural network characteristics, and all sociodemographic covariates. This finding suggests alignment with the NEM’s concept of *experiential homophily*, or the tendency for individuals to seek advice and information from network members who have undergone similar health experiences (27). When someone in one’s immediate social environment has used a DMHI, that visible engagement may serve as a form of social modeling (45), communicating that digital help-seeking is a viable, acceptable, and available option, and a signal that appears to meaningfully increase the likelihood of intending to do the same.

Being *engaged in formal treatment* appears to increase DMHI intention substantially, likely through several complementary NEM-consistent mechanisms such as exposure to clinical recommendations of digital tools by providers, normalization of help-seeking as an ongoing rather than episodic behavior, and increased familiarity with the mental health care ecosystem. This finding reinforces the pattern observed in the companion analysis which was that DMHIs appear more likely to function as adjuncts or complements to existing care than as primary on-ramps for treatment-naive individuals.

Notably, *not knowing* if someone in one’s social network used DMHIs was not significantly associated with the likelihood to use a DMHI in main effects models. However, when exploring the interaction between awareness of friends and family use of DMHI and perceived public stigma, this relationship becomes more complex. Specifically, among individuals who were uncertain (did not know) whether their friends and family used DMHIs, *higher public stigma was associated with substantially greater DMHI intention*. This pattern was not observed among those who knew for sure if a network member used DMHIs, for whom the main effect of public stigma operated without meaningful moderation.

The finding for the “not knowing” group is counter to the main effect of higher perceived public stigma leading to lower likelihood of intending to use a DMHI. Instead, for the “not knowing” group, higher perceived public stigma is associated with a higher likelihood of using a DMHI. One possible interpretation is that AANHPI emerging adults who are unaware of others in their network who use DMHIs may themselves be open to trying DMHIs as a way to avoid stigmatization. In other words, “not knowing” if others in their networks use or do not use DMHIs may indicate a lack of discussion around DMHIs (and perhaps broader mental health). This ambiguity, combined with the fear of stigmatization from their network, may result in their turning to more “private” channels of support such as digital technology.

#### Network size, social support availability, and help-seeking

Contrary to hypothesis 2, family network size and friend network size were not found to have a statistically significant association with DMHI intention. However, this null finding should still be interpreted with caution. It is possible that family or friend networks exert their influence on DMHI intentions through other mechanisms reflected in other network-level characteristics such as friend or family use of DMHIs, levels of mental health stigma within individuals’ social networks, or the function of individuals’ social networks (e.g., emotional support vs. instrumental support). This is reflected somewhat in the finding above, that friends and family use of DMHI (capturing network behavior) is significantly associated with an intention to use DMHI. In AANHPI cultural contexts where family systems occupy a central role in health decision-making (7, 29), the behavior and attitudes of family members may be more influential than the size of the network.

Social support availability was also not significantly associated with DMHI intention, and thus we fail to reject the null hypothesis for H3. Similar to network size, social support availability is less directly associated with intention to use DMHIs than other network-level characteristics. Together, these findings suggest a refinement of the NEM’s application in the DMHI context. Specifically, perhaps what matters more at the network-level is the specific content and behaviors of one’s network, rather than merely the quantity and availability.

### Robustness of Individual-Level SMHC Predictors

The individual-level SMHC predictors established in the companion analysis proved robust to the inclusion of social network variables, suggesting that there is value to integrating individual-level and social network-level theories of help-seeking. Perceived need, mental health literacy, and public stigma each retained significance across Models 1 and 2 with minimal reduction in magnitude.

Symptom severity (i.e., the presence of clinical depression, anxiety, or both) remained non-significant across all models. This pattern is consistent with the SMHC models’ conceptualization of symptom severity as an antecedent to perceived need, rather than a direct determinant of help-seeking intention (19, 20). In other words, symptom severity alone is not predictive of or associated with the intention to use DMHIs. Instead, there is a need to account for variables such as stigma and mental health literacy before individuals deem it necessary to address symptoms of mental illness. This also aligns with the NEM’s conceptualization of mental illness as a social process of symptom interpretation, encouragement, and support, rather than something that is purely individual rational choice (24, 27). From this perspective, the non-significance of symptom severity is not a null finding but a theoretically meaningful finding that suggests social and attitudinal conditions that surround symptoms matter more for DMHI intention than the symptoms themselves.

Self-stigma is worth separate attention. As the models adjusted for social network variables, its association strengthened. This shift may be informative, suggesting that the positive association between self-stigma and DMHI intention becomes more apparent once social network factors are accounted for. One interpretation may be that individuals with higher internalized self-stigma may be especially drawn to the anonymity of digital tools as a means of avoiding the social exposure associated with traditional help-seeking. This is consistent with broader literature on stigma and mental health help-seeking (46) and DMHIs as ways to avoid stigma (10, 11).

### Limitations

Several limitations should be noted. First, the cross-sectional nature of the survey prevents any causal inferences with the observed associations. The pathway from social network exposure to DMHI intention is theorized as directional within the NEM framework, but the present data cannot rule out reverse causation or confounding by unmeasured variables. Second, the nature of recruitment from an online panel likely overrepresents digital engaged AANHPI emerging adults, limiting generalizability to less connected or more marginalized subgroups. Third, measurement of certain constructs necessitated simplifications that may affect construct validity. For example, the acculturation proxy captures only one dimension of a more complex, multifaceted construct (47, 48). The friends and family DMHI use variable relies on respondent awareness, and associations observed from the “not known” group is inherently complex. Fourth multicollinearity was notably high (VIF > 10) for several of the covariates and for social support availability and mental health literacy variables. To adhere to the central frameworks, we included all of the variables in our models. However, we acknowledge that this may introduce challenges in interpreting the effects of those variables, as the shared variance among these constructs means the fully adjusted models may underestimate the importance of these variables, even while individual subscales fail to reach significance. Finally, the substantial influence of Vendor 3 on outcomes introduces sampling-related variance that may affect the generalizability of certain findings, although we did control for vendor source in our main analysis. Future studies should employ probability-based or community-recruited sampling strategies that do not introduce additional confounders.

### Implications and Future Directions

Despite these limitations, the findings offer direction for future research and practice. The strong association between awareness of friends and family who have used DMHIs and the intention to use DMHIs suggests that network-level interventions and strategies (e.g., peer programs, educating friends and family, and embedding DMHIs recommendations within networks such as religious communities) may be effective approaches for promoting DMHI engagement among AANHPI emerging adults. The interaction between public stigma and a lack of awareness of peer DMHI further suggests that intervention strategies tailored to individuals without existing peer exposure may need to encourage conversations with peers centered around mental health. This may come in the form of stigma-reduction interventions such as personalized normative feedback (49) or through psychoeducational programs (50).

Future research should explore correlates of actual use of DMHIs, as intention is a strong but imperfect predictor of actual use. Furthermore, integrating other frameworks such as technology acceptance frameworks or culturally specific models of mental health help-seeking would allow for a deeper examination of the mechanisms shaping DMHI use among AANHPI emerging adults, and perhaps other underserved communities. Relatedly, community engaged group-model building approaches (51) could be particularly valuable and informative for developing a richer and more concise mapping of pathways for DMHI use among AANHPI emerging adults.

Qualitative and mixed-methods approaches would be particularly valuable for understanding the meaning attached to several key associations such as awareness of DMHI use within social networks and the social dynamics that are involved. More complex methodologies such as social network analysis (52) would be useful in understanding network-characteristics and network dynamics that lead to the intention to use DMHIs. Given the correlation between network support and support quality within AANHPI communities, more advanced analytical methods such as structural equation modeling (SEM) may help account for latent structures (53).

The non-significance of mental health symptomatology indicates that programs and interventions utilizing DMHIs should focus on building individuals’ ability to accurately recognize symptoms of mental illness, and the need to address them, rather than targeting symptom severity alone. This can be done through interventions that support mental health literacy (54) and build awareness of different treatment options. The non-significance of mental health symptomatology also alludes to the episodic nature of mental illness as noted by the NEM (24). This, combined with the strong association of DMHI intention with engagement in clinical services, indicates that DMHIs may be most valuable as a supportive tool that can address mental health needs between sessions, or as episodes of mental illness arise. Given the impact of social modeling, additional interventions should explore the use of “influence maximization” to identify key network influencers (52, 55) and training existing social supports to provide the necessary “cues” to normalize and encourage DMHI use.

## CONCLUSION

The integration of the SMHC model and the NEM provides a strong framework for understanding the drivers of DMHI adoption among AANHPI emerging adults. This study’s theoretical contribution moves the focus on DMHI adoption beyond individual-level cognitive factors to demonstrate that digital help-seeking also involves a largely social process, heavily influenced by health discussions, social modeling, and existing network structures. A primary contribution of this research is the finding that beyond individual attitudes and knowledge, awareness of DMHI use in one’s own social network can serve as a meaningful signal to use DMHIs as a legitimate, viable, and acceptable option, while a lack of awareness leads one to be influenced by their own perceptions of how the public perceives mental illness. This finding is consistent with the NEM’s emphasis on experiential homophily as a mechanism of action for people to seek support. Interestingly, general network size and overall availability of social support were not associated with intention to use DMHIs, suggesting that shared attitudes towards mental health care, and the purpose of someone’s personal network matter far more than size and availability of support.

Building from Bunyi et al. (19), this study continues to demonstrate that symptom severity is further removed as an antecedent to DMHI intention rather than a direct predictor. This suggests that symptom severity alone, without the attitudinal and social conditions that turn symptoms into perceived need, are insufficient to motivate digital help-seeking behavior. Instead, a perceived need for support continues to emerge as a primary driver, alongside network-level variables. This aligns with the integrated framework’s assertion that clinical distress must be recognized and socially validated before it triggers a pathway to care.

The implementation of digital interventions among AANHPI emerging adults should shift towards strategies that include targeting mental health literacy to aid in the accurate recognition of the need to address symptoms of mental illness. Further, interventions should leverage existing social networks through strategies such as influence maximization, training existing family and peer supports, or involving clinicians.

Future research must employ qualitative and mixed-methods approaches to deeply explore the mechanisms behind observed social modeling and experiential homophily. By understanding the lived experiences and unique cultural nuances among AANHPI ethnic groups, the field can continue to refine this emerging theoretical framework and design more equitable and culturally considered supports for those who seek help digitally.

## Supplementary Material

Supplementary Files

This is a list of supplementary files associated with this preprint. Click to download.

- APPENDICES.docx

## Figures and Tables

**Table 1:** Sample Characteristics by DMHI Intention Status (N = 1,577)

Characteristic	Overall % or M (SD)	No DMHI Intent % or M (SD)	DMHI Intent % or M (SD)	p	Effect size
**Outcome**					
DMHI intention (yes), n (%)	1577	669	908		
**Sociodemographics**					
Age, M (SD)	23.95 (3.30)	23.10 (3.41)	24.57 (3.07)	p < .001	d = 0.46
Sex (female), n (%)	763 (48.4%)	357 (53.4%)	406 (44.7%)	p < .01	φ = 0.09
College degree or higher, n (%)	967 (61.3%)	325 (48.6%)	642 (70.7%)	p < .001	φ = 0.22
Currently employed, n (%)	926 (58.7%)	335 (50.1%)	591 (65.1%)	p < .001	φ = 0.15
Health insurance (insured), n (%)	1454 (92.2%)	590 (88.2%)	864 (95.2%)	p < .001	φ = 0.13
High acculturation, n (%)	935 (59.3%)	315 (47.1%)	620 (68.3%)	p < .001	φ = 0.21
Ethnicity, n (%)				p < .001	V = 0.33
Chinese (reference)	198 (12.5%)	120 (18.0%)	77 (8.5%)		
Filipino	165 (10.5%)	72 (10.8%)	93 (10.2%)		
Indian	197 (12.5%)	85 (12.7%)	112 (12.3%)		
Japanese	147 (9.3%)	17 (2.5%)	130 (14.3%)		
Korean	150 (9.5%)	56 (8.4%)	94 (10.4%)		
NHPI	185 (11.7%)	29 (4.3%)	156 (17.2%)		
Vietnamese	138 (8.8%)	79 (11.8%)	59 (6.5%)		
Self-Described AANHPI	136 (8.6%)	69 (10.3%)	67 (7.4%)		
Multi-ethnic	54 (3.4%)	34 (5.1%)	20 (2.2%)		
Multi-racial AANHPI	208 (13.2%)	108 (16.1%)	100 (11.0%)		
**SMHC Predictors**					
Perceived need for support (yes), n (%)	807 (51.2%)	226 (33.8%)	581 (64.0%)	p < .001	φ = 0.30
Mental health literacy score, M (SD)	5.19 (1.87)	4.78 (1.94)	5.50 (1.77)	p < .001	d = 0.39
Self-stigma, M (SD)	1.09 (0.52)	1.01 (0.52)	1.15 (0.50)	p < .001	d = 0.27
Public stigma, M (SD)	1.49 (0.39)	1.52 (0.43)	1.46 (0.36)	p < .01	d = 0.15
Symptom Severity, n (%)				p < .001	V = 0.24
No clinical symptoms	645 (40.9%)	352 (52.6%)	293 (32.3%)		
Depression-only	170 (10.8%)	78 (11.7%)	92 (10.1%)		
Anxiety-only	63 (4.0%)	34 (5.1%)	29 (3.2%)		
Both depression & anxiety	699 (44.3%)	205 (30.6%)	494 (54.4%)		
**Network Episode Model Predictors**					
Friends/family DMHI use, n (%)				p < .001	V = 0.30
Yes	625 (39.6%)	158 (23.6%)	467 (51.4%)		
Not known	287 (18.2%)	179 (26.8%)	108 (11.9%)		
No (reference)	665 (42.2%)	332 (49.6%)	333 (36.7%)		
LSNS-6 Family subscale, M (SD)	7.16 (3.80)	6.03 (3.49)	7.99 (3.81)	p < .001	d = 0.54
LSNS-6 Friend subscale, M (SD)	7.66 (3.85)	6.80 (3.79)	8.30 (3.77)	p < .001	d = 0.40
MOS Total score, M (SD)	14.16 (6.20)	12.70 (6.81)	15.25 (5.46)	p < .001	d = 0.41
Currently in therapy (yes), n (%)	464 (29.4%)	85 (12.7%)	379 (41.7%)	p < .001	φ = 0.31
**Other**					
Vendor source, n (%)				p < .001	V = 0.39
Vendor 1 (reference)	713 (45.2%)	380 (56.8%)	333 (36.7%)		
Vendor 2	490 (31.1%)	260 (38.9%)	230 (25.3%)		
Vendor 3	374 (23.7%)	29 (4.3%)	345 (38.0%)		

Abbreviations. DMHI = digital mental health intervention; SMHC = Seeking Mental Health Care model; LSNS-6 = Lubben Social Network Scale-6; MOS = Medical Outcomes Study Social Support Survey; NHPI = Native Hawaiian/Pacific Islander; AANHPI = Asian American, Native Hawaiian, and Pacific Islander; GAD-7 = Generalized Anxiety Disorder 7-item scale; PHQ-9 = Patient Health Questionnaire 9-item scale; M = mean; SD = standard deviation.

Effect sizes: Cohen’s d reported for continuous variables (small ≥ 0.20, medium ≥ 0.50, large ≥ 0.80); phi (φ) reported for binary categorical variables; Cramér’s V reported for multicategory categorical variables.

p-values derived from independent samples t-tests (continuous variables) and chi-square tests (categorical variables).

**Table 2.** Model 1: SMHC-Only Logistic Regression Predicting DMHI Intention (N = 1,577) Symptom severity operationalized as four-category variable (None / Depression only / Anxiety only / Both)

Variable	AOR	SE	LL	UL
**SMHC Predictors**				
Perceived need (yes)	1.94***	0.26	1.49	2.53
Mental health literacy	1.14***	0.04	1.07	1.22
Self-stigma	1.38*	0.18	1.07	1.78
Public stigma	0.60**	0.09	0.44	0.81
**Symptom Severity (ref: No clinical symptoms)**				
Depression only	1.01	0.20	0.69	1.48
Anxiety only	0.68	0.20	0.38	1.22
Both	1.12	0.17	0.84	1.50
**Covariates**				
Female sex	1.15	0.15	0.89	1.48
Age	1.05*	0.02	1.01	1.09
College degree or higher	1.36*	0.19	1.03	1.79
Currently employed	0.89	0.12	0.69	1.15
Insured	1.70*	0.36	1.12	2.59
High acculturation	1.42**	0.18	1.11	1.82
Currently in therapy	2.18***	0.35	1.59	2.99
**Ethnicity (ref: Chinese)**				
Filipino	1.75*	0.41	1.11	2.76
Indian	1.79**	0.39	1.16	2.76
Japanese	3.41***	1.18	1.73	6.72
Korean	0.98	0.26	0.58	1.66
NHPI	1.57	0.50	0.84	2.94
Vietnamese	1.15	0.28	0.72	1.84
Self-Described AANHPI	1.77*	0.43	1.09	2.87
Multi-ethnic	0.76	0.26	0.39	1.50
Multi-racial AAPI	1.13	0.26	0.72	1.76
**Vendor (ref: Vendor 1)**				
Vendor 2	0.88	0.11	0.68	1.14
Vendor 3	3.82***	1.04	2.24	6.52

LR χ^2^(25) = 466.35, p < .001 | Pseudo R^2^ = 0.217 | Log-likelihood = −841.74

Note. AOR = adjusted odds ratio; SE = standard error; LL/UL = lower/upper limit of 95% confidence interval; FF = friends and family; LSNS-6 = Lubben Social Network Scale-6; MOS = Medical Outcomes Study Social Support Survey; MHL = mental health literacy; SMHC = Seeking Mental Health Care model; NEM = Network Episode Model. Symptom severity categories based on PHQ-9 ≥ 10 (depression) and GAD-7 ≥ 10 (anxiety) clinical cutoffs.

* p < .05

** p < .01.

*** p < .001.

**Table 3.** Model 2: SMHC + NEM Main Effects Logistic Regression Predicting DMHI Intention (N = 1,577)

Variable	AOR	SE	LL	UL
**NEM Predictors**				
FF use DMHI (ref: No)				
FF use DMHI (Yes)	1.79***	0.26	1.35	2.39
FF use DMHI (Not known)	0.75	0.12	0.55	1.03
LSNS-6 Family	1.03	0.02	0.99	1.08
LSNS-6 Friend	0.99	0.02	0.95	1.03
MOS Total	1.02	0.01	0.99	1.04
Currently in therapy	1.84***	0.31	1.33	2.55
**SMHC Predictors**				
Perceived need (yes)	1.91***	0.26	1.46	2.50
Mental health literacy	1.11**	0.04	1.03	1.18
Self-stigma	1.41*	0.19	1.09	1.84
Public stigma	0.63**	0.10	0.46	0.85
**Symptom Severity (ref: No clinical symptoms)**				
Depression only	1.05	0.21	0.71	1.55
Anxiety only	0.67	0.20	0.37	1.20
Both	1.14	0.17	0.85	1.53
**Covariates**				
Female sex	1.13	0.15	0.87	1.46
Age	1.05*	0.02	1.01	1.09
College degree or higher	1.28	0.18	0.97	1.70
Currently employed	0.85	0.11	0.66	1.10
Insured	1.70*	0.37	1.11	2.60
High acculturation	1.42**	0.18	1.11	1.83
**Ethnicity (ref: Chinese)**				
Filipino	1.85**	0.44	1.16	2.94
Indian	1.77*	0.40	1.14	2.74
Japanese	3.60***	1.26	1.81	7.16
Korean	0.93	0.25	0.55	1.59
NHPI	1.42	0.46	0.75	2.70
Vietnamese	1.12	0.27	0.69	1.81
Self-Described AANHPI	1.73*	0.43	1.06	2.83
Multi-ethnic	0.72	0.25	0.37	1.42
Multi-racial AAPI	1.10	0.26	0.70	1.73
**Vendor (ref: Vendor 1)**				
Vendor 2	0.88	0.12	0.68	1.14
Vendor 3	3.28***	0.91	1.90	5.65

LR χ^2^(30) = 506.31, p < .001 | Pseudo R^2^ = 0.236 | Log-likelihood = −821.76 | LR test vs. Model 1: χ^2^(5) = 39.96, p < .001

Note. AOR = adjusted odds ratio; SE = standard error; LL/UL = lower/upper limit of 95% confidence interval; FF = friends and family; LSNS-6 = Lubben Social Network Scale-6; MOS = Medical Outcomes Study Social Support Survey; MHL = mental health literacy; SMHC = Seeking Mental Health Care model; NEM = Network Episode Model. Symptom severity categories based on PHQ-9 ≥ 10 (depression) and GAD-7 ≥ 10 (anxiety) clinical cutoffs.

* p < .05

** p < .01.

*** p < .001.

**Table 4.** Model 3c — FF DMHI Use × Public Stigma

Variable	AOR	SE	LL	UL
**NEM Predictors**				
FF use DMHI (ref: No)				
FF use DMHI (Yes)	1.78***	0.26	1.33	2.37
FF use DMHI (Not known)	0.75	0.12	0.54	1.03
LSNS-6 Family	1.03	0.02	0.99	1.08
LSNS-6 Friend	0.99	0.02	0.95	1.03
MOS Total	1.02	0.01	0.99	1.04
Currently in therapy	1.86***	0.31	1.34	2.58
**Interaction Terms (ref: FF use DMHI = No)**				
FF use DMHI (Yes) × Public Stigma	0.79	0.27	0.41	1.53
FF use DMHI (Not known) × Public Stigma	3.12** †	1.21	1.46	6.66
**SMHC Predictors**				
Perceived need (yes)	1.94***	0.27	1.48	2.53
Mental health literacy	1.11**	0.04	1.03	1.18
Self-stigma	1.43**	0.19	1.10	1.86
Public stigma (mean-centered)	0.52**	0.12	0.34	0.82
**Symptom Severity (ref: No clinical symptoms)**				
Depression only	1.06	0.21	0.72	1.57
Anxiety only	0.64	0.19	0.36	1.16
Both	1.13	0.17	0.84	1.52
**Covariates**				
Female sex	1.12	0.15	0.86	1.45
Age	1.05*	0.02	1.01	1.09
College degree or higher	1.25	0.18	0.94	1.66
Currently employed	0.86	0.11	0.66	1.11
Insured	1.72*	0.37	1.12	2.63
High acculturation	1.42**	0.18	1.10	1.83
**Ethnicity (ref: Chinese)**				
Filipino	1.88**	0.45	1.18	3.00
Indian	1.70*	0.38	1.09	2.64
Japanese	3.65***	1.29	1.83	7.29
Korean	0.92	0.25	0.54	1.58
NHPI	1.39	0.46	0.73	2.66
Vietnamese	1.11	0.27	0.69	1.80
Self-Described AANHPI	1.70*	0.43	1.04	2.79
Multi-ethnic	0.68	0.24	0.34	1.34
Multi-racial AAPI	1.06	0.25	0.67	1.68
**Vendor (ref: Vendor 1)**				
Vendor 2 (PureSpectrum)	0.90	0.12	0.70	1.17
Vendor 3 (Torfac)	3.27***	0.91	1.90	5.65

LR χ^2^(32) = 518.96, p < .001 | Pseudo R^2^ = 0.241 | Log-likelihood = −815.43 | LR test vs. Model 2: χ^2^(2) = 12.65, p = .002

Note. AOR = adjusted odds ratio; SE = standard error; LL/UL = lower/upper limit of 95% confidence interval; FF = friends and family; LSNS-6 = Lubben Social Network Scale-6; MOS = Medical Outcomes Study Social Support Survey; MHL = mental health literacy; SMHC = Seeking Mental Health Care model; NEM = Network Episode Model. Symptom severity categories based on PHQ-9 ≥ 10 (depression) and GAD-7 ≥ 10 (anxiety) clinical cutoffs.

* p < .05

** p < .01.

*** p < .001

† = Adjusted p-value (Bonferroni correction, significant at α = 0.017)

**Table 5.** Model Fit Statistics

Model	df	Log-likelihood	Overall LR χ^2^ (df)	Pseudo-R^2^	AIC	BIC	ΔAIC vs. Model 2	ΔBIC vs. Model 2	LR χ^2^ vs. Model 2 (df)
Model 1: SMHC predictors only	25	−841.74	466.35 (25)***	0.217	1,735.47	1,874.92	—	—	—
Model 2: SMHC + NEM predictors	30	−821.76	506.31 (30)***	0.236	1,705.52	1,871.78	—	—	—
Model 3a: Model 2 + Perceived Need × LSNS-6 Family	31	−820.50	508.82 (31)***	0.237	1,705.01	1,876.64	−0.51	+4.86	2.51 (1), p = .113
Model 3b: Model 2 + MHL × Friends/Family DMHI Use	32	−820.03	509.77 (32)***	0.237	1,706.05	1,883.04	+0.53	+11.26	3.46 (2), p = .177
Model 3c: Model 2 + Public Stigma × Friends/Family DMHI Use	32	−815.43	518.96 (32)***	0.241	1,696.87	1,873.86	−8.65	+2.08	12.65 (2), p = .002**

Note. SMHC = Seeking Mental Health Care model. NEM = Network Episode Model. MHL = Mental Health Literacy. DMHI = Digital Mental Health Intervention. LSNS-6 = Lubben Social Network Scale-6. LR = Likelihood Ratio. AIC = Akaike’s Information Criterion. BIC = Bayesian Information Criterion. Δ columns reflect difference relative to Model 2. Overall LR χ^2^ tests model against null (intercept-only). df in parentheses for LR χ^2^ columns. All models: N = 1,577.

** p < .01.

*** p < .001.

## Data Availability

The datasets used and/or analyzed during the current study are available from the corresponding author on reasonable request.

## Abbreviations

AANHPI: Asian American, Native Hawaiian, and Pacific Islander
DMHI: Digital Mental Health Intervention
SMHC: Seeking Mental Health Care (Model)
NEM: Network Episode Model
MHL: Mental Health Literacy
PHQ-9: Patient Health Questionnaire, 9-item
GAD-7: Generalized Anxiety Disorder Scale, 7-item
LSNS-6: Lubben Social Network Scale, 6-item
MOS: Medical Outcomes Study Social Support Survey (also referenced as MOS-SSS)
PDDS: Perceived Devaluation and Discrimination Scale
CHIS: California Health Interview Survey
PNF: Personalized Normative Feedback (Project)
IRB: Institutional Review Board
NHPI: Native Hawaiian and Pacific Islander
AOR: Adjusted Odds Ratio
OR: Odds Ratio
CI: Confidence Interval
AIC: Akaike Information Criterion
BIC: Bayesian Information Criterion
LR: Likelihood Ratio
VIF: Variance Inflation Factor
SEM: Structural Equation Modeling
FF: Friends and Family
SD: Standard Deviation
NCATS: National Center for Advancing Translational Science
NIH: National Institutes of Health
